# The Relationship Between Serum Selenium Level, Cognitive Functions, and Depression in Patients With Chronic Kidney Disease

**DOI:** 10.7759/cureus.37233

**Published:** 2023-04-06

**Authors:** Duygu Tutan, Barış Eser, Ibrahim Dogan, Nihal Aydemir, Huseyin Kayadibi

**Affiliations:** 1 Department of Internal Medicine, Erol Olçok Research and Training Hospital, Çorum, TUR; 2 Department of Nephrology, Hitit University Faculty of Medicine, Çorum, TUR; 3 Department of Biochemistry, Eskişehir Osmangazi University Faculty of Medicine, Eskişehir, TUR

**Keywords:** selenium, dialysis, depression, cognitive impairment, chronic kidney disease

## Abstract

Introduction

Impairment of cognitive functions can commonly develop in patients with chronic kidney disease (CKD) and increase morbidity and mortality. The antioxidant activity of selenium reduces cognitive decline by protecting neurons from free radical damage. We aimed to explore the associations between serum selenium levels, cognitive impairment, and depression in CKD patients in this research.

Methods

In this prospective cross-sectional research, 100 participants between the ages of 20 and 65 were included, and four groups of 25 patients each were formed (control group, stage 3-4 CKD, peritoneal dialysis [PD], hemodialysis [HD]). The Standardized Mini Mental Test (sMMT) was used to measure cognitive skills, and the Beck Depression Inventory (BDI) was utilized to diagnose depression. Simultaneously, measurements of serum selenium levels were done from collected blood samples.

Results

Cognitive impairment was detected in 4% of the control group, 16% of CKD patients (n=75), and 30% of the dialysis patients (n=50). Depression was found in 16% of the control group, 40% of the stage 3-4 CKD group, 50% of the PD group, and 44% of the HD group. In the control group, sMMT scores were higher than the other groups (p<0.001 for all), while the BDI score was statistically significantly lower (p=0.003). Serum selenium levels were found to be higher than HD and PD groups in patients with non-dialysis CKD and control groups in the post hoc analyses (p=0.001, p<0.001, p<0.001, p<0.001, respectively).

Conclusion

Depression and cognitive impairment are particularly prevalent in CKD and dialysis patients. Our results indicate serum selenium insufficiency may be related to depression and cognitive impairment in this patient group. Nonetheless, these findings need to be confirmed by larger-scale studies.

## Introduction

Chronic kidney disease (CKD) is characterized by an irreversible, progressive decline in kidney function, and is an important public health problem affecting the quality of life, morbidity, and mortality [1]. It is known that cognitive impairment, which is reported to develop in 16-40% of CKD patients and up to 60% of dialysis patients, also decreases the quality of life, decision-making ability, diet, and treatment compliance, while increasing the need and duration of hospitalization, and consequently the morbidity and mortality. The etiology of cognitive impairment in this patient population has not been fully elucidated and is considered multifactorial. However, concomitant diabetes, hypertension (HT), dyslipidemia, and uremic toxins are also thought to affect cognitive impairment [2].

It has been shown that selenium can influence the antioxidant process and neuromodulation against oxidative stress. It has been suggested that selenium may slow down cognitive deterioration by preventing the damage caused by free radicals in neurons [3]. Dietary selenium intake was also low in those with high cognitive impairment [4]. On the other hand, hemodialysis (HD) patients had a significantly lower serum selenium level (SSL) compared to healthy volunteers [5].

Many psychiatric and psychosocial problems are encountered during the course of CKD. Psychiatric disorders are associated with increased morbidity, mortality, frequency of hospitalization, and health expenditures in end-stage renal disease (ESRD) patients [6]. There is a dilemma regarding the effects of selenium on depression. In some studies, it was found that low dietary selenium intake may be a high-risk factor for the development of major depression [7], while some studies did not find any significant relationship between low selenium and depression in HD patients [8]; in contrast, some studies suggested that high selenium exposure is associated with severe depression symptoms [9]. Because of this inconsistency, more studies are needed to clarify the relationship of selenium with depression.

To the best of our knowledge, there are no published studies examining the associations between CKD patients’ blood selenium levels and cognitive impairment and depression. However, it is known that depression may also affect cognitive functions [10]. Therefore, this study aimed to contribute to the literature by evaluating the effect of serum selenium on cognitive performance and depression in CKD patients.

## Materials and methods

The study was started after the approval of the Hitit University Research Ethics Committee (Date/No: 08.01.2020/147). Participants were given detailed information about the study, and written consent was obtained in accordance with the Declaration of Helsinki.

A total of 100 participants followed in the nephrology clinic and dialysis unit between the ages of 20 and 65 were included in this controlled cross-sectional study. Volunteers were categorized into four groups: Group 1 as HD treatment patients, Group 2 as patients with continuous peritoneal dialysis (PD), Group 3 as patients with stage 3-4 CKD (glomerular filtration rate [GFR]: 59-15 mL/dL/1.73 m^2^), Group 4 as non-CKD patients. Participants in the dialysis treatment group had received HD or PD treatment for at least six months.

Those who do not have any known diagnosis of cerebrovascular disease, major depression, dementia, acute or chronic active infections, active malignancy, pregnancy, thyroid dysfunction, auditory and visual problems that may cause participants to have difficulty performing the Standardized Mini Mental Test (sMMT) and depression assessment tests, and individuals who can speak in the native language and are literate were included. Patients using any multivitamin or trace element supplements were also excluded. Somatometric and demographic characteristics of the participants, laboratory tests used in routine follow-up, accompanying additional diseases, medications, and habits were recorded. The education levels of the participants were questioned and recorded as primary school (1-5 years), middle school (6-8 years), high school (9-12 years), and university (over 12 years).

After overnight fasting, complete blood count, glucose, blood urea nitrogen, creatinine, GFR, albumin, calcium, phosphorus, uric acid, parathormone, ferritin, bicarbonate, lipid profile, and C-reactive protein (CRP) levels were analyzed. Blood samples were taken before the middle dialysis session of the week for HD patients, at outpatient clinic control for PD patients, and at the time of inclusion for the control group. Estimated glomerular filtration rate (eGFR) values were determined using the Chronic Kidney Disease Epidemiology Collaboration equation [11]. Kt/V_urea_ was calculated to evaluate HD and PD qualifications.

The blood sample taken into the vacutainer tube was kept at room temperature for 30 minutes, then centrifuged at 1.310 *g* for 10 minutes, and the separated serum was stored at -80 °C until analysis. Measurement of SSL was done by mass spectrometry method in ICP-MS (Agilent-7500) device [12].

Application and interpretation of the sMMT

In the week following the collection of blood samples, sMMT was applied to HD patients before the mid-week dialysis session and PD patients under outpatient control to evaluate cognitive functions.

sMMT assesses orientation, memory, attention, language, and visuospatial skills. It consists of 10 points of time and space orientation, 3 points of recording, 3 points of recall, 6 points of memory, 5 points of attention, 8 points of language, and 1 point of visual-spatial functions, as a total of 30 points. Normal cognitive function is defined as at least 24 points, while 23 or fewer points as dysfunction in sMMT [13].

Application and interpretation of the Beck Depression Inventory

Since cognitive functions may be affected in the presence of depression, the Beck Depression Inventory (BDI) was applied to evaluate depression simultaneously with the application of the sMMT.

It has been shown that the BDI can be applied reliably to detect symptoms of depression in people with CKD [14]. Using this inventory, we aimed to objectively determine the degree of depression symptoms in patients. The BDI is a 21-item self-report rating inventory that assesses depression-related attitudes and symptoms. BDI’s sub-groups are emotion, pessimism, the feeling of failure, joylessness, guilt, punishment, self-dislike, self-blame, suicidal thoughts, crying, anger, social withdrawal, instability, change in body image, working disability, sleep disturbance, fatigue, anorexia, weight loss, somatic complaints, and loss of libido. Each item on the inventory was scored between 0 and 3. A total score between 11 and 17 was determined to indicate mild depression, between 18-29 was determined to indicate moderate depression, and between 30-63 was determined to indicate severe levels of depression [15].

Statistical analysis

The SPSS package software (version 22.0, SPSS Inc, Chicago, IL, USA) was used for the statistical analyses. For normally distributed continuous data, the descriptive statistics were reported as mean ± standard deviation; for non-normally distributed continuous data, the median (min-max) was used; and for categorical data, the numbers and percentages (%) were used. The normality distribution was analyzed by the Shapiro-Wilk test. In the comparison of continuous variables between two independent groups, the student’s t-test was used for normally distributed variables in independent groups, and the Mann-Whitney U test was used for variables not normally distributed. One-way analysis of variance (ANOVA) was used for normally distributed variables, and the Kruskal-Wallis test was used for variables not normally distributed in comparing continuous variables among more than two independent groups. For identifying the cause of the difference in ANOVA and Kruskal Wallis tests, multiple Mann-Whitney U post hoc comparison tests with Tukey or Bonferroni correction were used. Relationships between categorical variables were investigated with either the chi-square test or Fisher's exact test, depending on the number of data in the crosstab cells. Depending on the data distribution, Pearson's or Spearman's correlation coefficient was used to assess the relationships between numerical variables. The cause-and-effect relationship between two numerical variables with strong correlations was determined using simple linear regression analysis. The statistical significance level was determined as p <0.05.

## Results

The mean age of 100 participants (53 men, 53%) was 48±11 years, and the male-female ratios and mean ages were statistically similar between the groups (p=0.066, p=0.161, respectively). Table 1 displays demographic information and general characteristics of all patients in the study. A statistically significant relationship was found between education level and residence between research groups (p<0.001, p=0.002, respectively).

**Table 1:** Comparison of the general characteristics of the research groups ^a^Chi-square test, ^b^Fisher exact test. CAD: coronary artery disease; CHF: congestive heart failure; BMI: body mass index; SBP: systolic blood pressure; DBP: diastolic blood pressure. Categorical variables were presented as frequency and percentage, and continuous variables as median, min-max, and mean±standard deviation according to distribution characteristics.

Variables		Group 1 (n=25)	Group 2 (n=25)	Group 3 (n=25)	Group 4 (n=25)	p-Value
Gender, n (%)	Male	16 (64)	17 (68)	9 (36)	11 (44)	0.066^a^
	Female	9 (36)	8 (32)	16 (64)	14 (56)	
Level of education, n (%)	Literate	0 (0)	1 (4)	1 (4)	0 (0)	<0.001^b^
	Primary	15 (60)	14 (56)	13 (52)	3 (12)	
	Middle	3 (12)	3 (12)	3 (12)	0 (0)	
	High school	4 (16)	6 (24)	5 (20)	6 (24)	
	University	3 (12)	1 (4)	3 (12)	16 (64)	
Age		49±10	51±11	49±13	44±10	0.161^a^
Diabetes, n (%)		2 (8)	10 (40)	6 (24)	3 (12)	0.025^a^
Hypertension, n (%)		21 (84)	24 (96)	23 (92)	3 (12)	<0.001^a^
CAD, CHF, n (%)		6 (24)	8 (32)	4 (16)	3 (12)	0.314^a^
Smoking, n (%)		7 (28)	2 (8)	4 (16)	9 (36)	0.080^a^
Alcohol, n (%)		1 (4)	0 (0)	0 (0)	1 (4)	1.000^b^
BMI, kg/m^2^		23.5±4.69	27.2±6.86	28.2±7.57	27.7±5.17	0.021^b^
SBP, mmHg		121±21	137±25	126±21	119±14	0.018^b^
DBP, mmHg		75±13	80±10	79±13	76±8	0.298^b^

**Figure 1:**
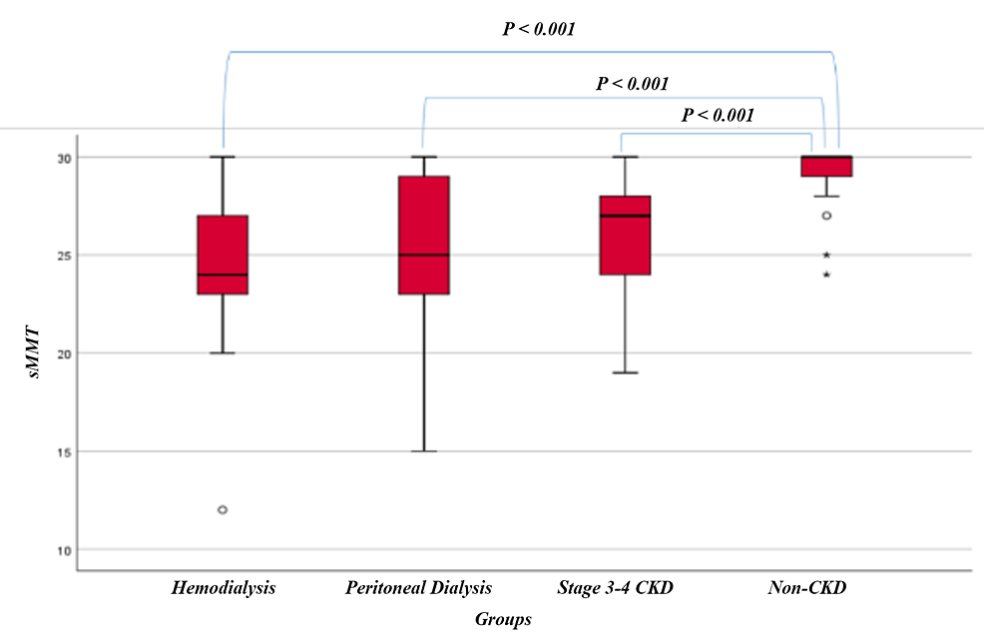
Comparison of sMMT scores among research groups sMMT: Standardized Mini Mental Test; CKD: chronic kidney disease.

**Figure 2:**
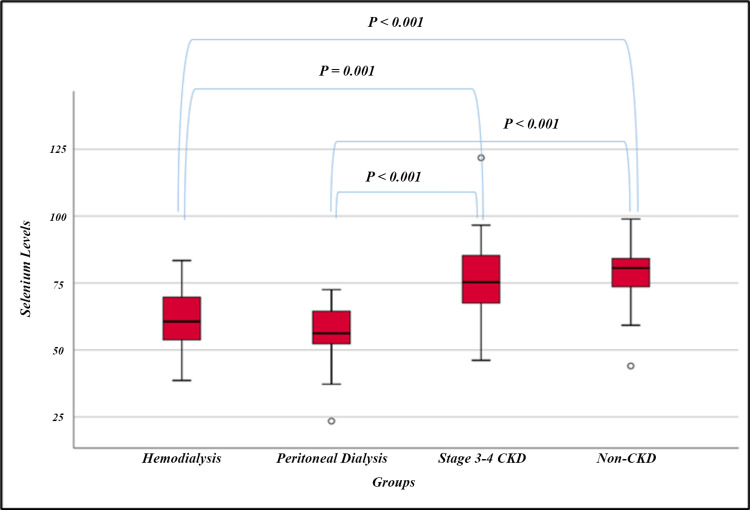
Comparison of serum selenium levels among research groups CKD: chronic kidney disease.

sMMT scores were statistically significantly higher in the non-CKD group than in the HD, PD, and stage 3-4 CKD groups (p<0.001 for all). sMMT scores were similar between HD and PD and stage 3-4 CKD groups (p=1.000, for both). sMMT scores were similar between PD and stage 3-4 CKD groups (p=1.000). BDI scores in the HD and PD groups were statistically significantly higher than in the non-CKD group (p=0.011, p=0.003, respectively). BDI scores were similar between HD and PD and stage 3-4 CKD groups (p=1.000, for both) (Table 2). SSLs were statistically significantly different between the groups (p<0.001). According to the post hoc analysis results, the SSLs of stage 3-4 CKD and non-CKD groups were statistically significantly higher than the SSLs of the HD and PD groups (p=0.001, p<0.001, p<0.001, p<0.001, respectively) (Table 3, Figure 1, Figure 2).

**Table 2:** Comparison of sMMT scores and BDI scores among research groups sMMT: Standardized Mini Mental Test; BDI: Beck Depression Inventory. Continuous variables were reported as median and min-max according to distribution characteristics.

Variables	Group 1	Group 2	Group 3	Group 4	p-Value	Post hoc p-value
sMMT (total) (0-30 points)	24 (12-30)	25 (15-30)	27 (19-30)	30 (24-30)	<0.001	1-4: <0.001
						2-4: <0.001
						3-4: <0.001
Orientation (0-10 points)	9 (6-10)	10 (4-10)	10 (7-10)	10 (9-10)	0.001	1-4: 0.001
						2-4: 0.046
						3-4: 0.014
Registration (0-3 points)	3 (1-3)	3 (3-3)	3 (3-3)	3 (1-3)	0.568	-
							Attention and calculation (0-5 points)	3 (0-5)	3 (0-5)	3 (0-5)	5 (0-5)	<0.001	1-4: 0.002
2-4: 0.004												
3-4: 0.003												
Recall (0-3 points)	2 (0-3)	2 (0-3)	2 (1-3)	3 (1-3)	0.019	2-4: 0.039
							Language (0-9 points)	7 (4-9)	8 (5-9)	9 (6-9)	9 (7-9)	<0.001	1-3: 0.011
1-4: <0.001												
BDI score (total) (0-63 points)	15 (0-52)	17 (2-34)	12 (2-27)	6 (0-36)	0.002	1-4: 0.011
						2-4: 0.003

**Table 3:** Comparison of laboratory values between research groups ^a^One-way ANOVA, ^b^Kruskal Wallis, ^c^Mann-Whitney U test. ANOVA: analysis of variance; BUN: blood urea nitrogen; LDL: low-density lipoprotein; CRP: C-reactive protein; TSH: thyroid-stimulating hormone; Ref: Reference.

Variables	n	Group 1 median (min-max) (mean±SD)	Group 2 median (min-max) (mean±SD)	Group 3 median (min-max) (mean±SD)	Group 4 median (min-max) (mean±SD)	p-Value	Post hoc p-value
								BUN, mg/dL (Ref: 3.73-23)	100	69±16	46±15	34±16	12±3	<0.001^a^	1-2: <0.001
															1-3: <0.001
1-4: <0.001														
2-3: 0.011														
2-4: <0.001														
3-4: <0.001														
Creatinine, mg/dL (Ref: 0.6-1.3)	100	9.8 (5.6-14.5)	7.5 (3.3-11.2)	1.9 (1.2-7.1)	0.8 (0.5-0.9)	<0.001^b^	1-3: <0.001
							1-4: <0.001
							2-3: 0.002
							2-4: <0.001
							3-4: 0.010
Glucose, mg/dL (Ref: 70-110)	100	90 (67-190)	100 (60-360)	94 (40-175)	93 (73-187)	0.126^b^	-
Triglyceride, mg/dL (Ref: 40-150)	98	149 (64-698)	150 (80-530)	135 (62-280)	143 (47-270)	0.829^b^	-
LDL cholesterol, mg/dL (Ref: 0-160)	97	86±28	119±56	126±44	127±30	0.002^a^	1-2: 0.029
							1-3: 0.006
							1-4: 0.004
Uric acid, mg/dL (Ref: 3.5-7.2)	100	6.74±1.12	5.77±1.60	6.69±1.69	5.28±1.29	0.001^a^	1-4: 0.003
							3-4: 0.005
Sodium, mEq/L (Ref: 136-146)	100	139 (130-142)	138 (129-142)	140 (134-145)	140 (134-144)	<0.001^b^	1-4: 0.011
							2-3: 0.021
							2-4: 0.001
Potassium, mEq/L (Ref: 3.5-5.5)	100	5.6 (3.7-8)	4.5 (3.4-5.8)	4.8 (3.5-5.7)	4.4 (3.9-5.4)	<0.001^b^	1-2: <0.001
							1-3: 0.044
							1-4: <0.001
Calcium, mg/dL (Ref: 8.6-10.2)	100	8.5 (7.2-10.3)	8.5 (7.4-10)	9 (8.3-10.2)	9.3 (8.9-10.4)	<0.001^b^	1-4: <0.001
							2-4: <0.001
Phosphorus, mg/dL (Ref: 2.5-4.5)	100	5.1 (2.9-8.3)	4.5 (2.5-6.3)	3.5 (2.4-5)	3.4 (2.6-4.4)	<0.001^b^	1-3: <0.001
							1-4: <0.001
							2-3: 0.010
							2-4: 0.003
Hemoglobin, g/dL (Ref: 13.5-16.9)	100	10.7 (8.5-13)	10.8 (7.5-14.2)	11.5 (9.8-16)	14.7 (8.3-16.9)	<0.001^b^	1-4: <0.001
							2-4: <0.001
							3-4: 0.004
Neutrophil/lymphocyte ratio (Ref: 0.78-3.53)	100	2.57 (0.973-9.20)	3.10 (0.842-7.44)	2.46 (1.03-9.36)	1.56 (0.846-5.12)	<0.001^b^	1-4: <0.001
							2-4: <0.001
							3-4: 0.005
CRP, mg/dL (Ref: 0-5)	98	7 (0.9-31)	6 (3-26)	3 (3-67)	3 (3-15)	0.018^b^	1-4: 0.019
Parathormone, pg/mL (Ref: 15-65)	94	540 (1-1494)	290 (1-1660)	154 (15-776)	38 (5-968)	<0.001^b^	1-3: <0.001
							1-4: <0.001
							2-3: 0.035
							2-4: <0.001
Ferritin level, ng/mL (Ref: 13-150)	100	653 (57-2000)	410 (103-1227)	63 (5-567)	48 (6-510)	<0.001^b^	1-3: <0.001
							1-4: <0.001
							2-3: <0.001
							2-4: <0.001
Vitamin B12 level, pg/mL (Ref. 191-663)	91	565 (150-1290)	425 (240-2000)	360 (140-2000)	313.5 (134-630)	0.033^b^	1-4: 0.041
TSH, µU/mL (Ref: 0.27-4.1)	88	1.30 (0.33-5.70)	2.20 (0.80-8.70)	1.65 (0.60-5.30)	2 (0.30-4.5)	0.550^b^	-
Serum selenium level, µg/L (Ref: 63-160)	100	62±13	57±12	76±15	78±12	<0.001^a^	1-3: 0.001
							1-4: <0.001
							2-3: <0.001
							2-4: <0.001
Kt/V_urea_	50	1.42 (0.90-2.51)	2.18 (1.39-4.93)	-	-	<0.001^c^	-

**Figure 3:**
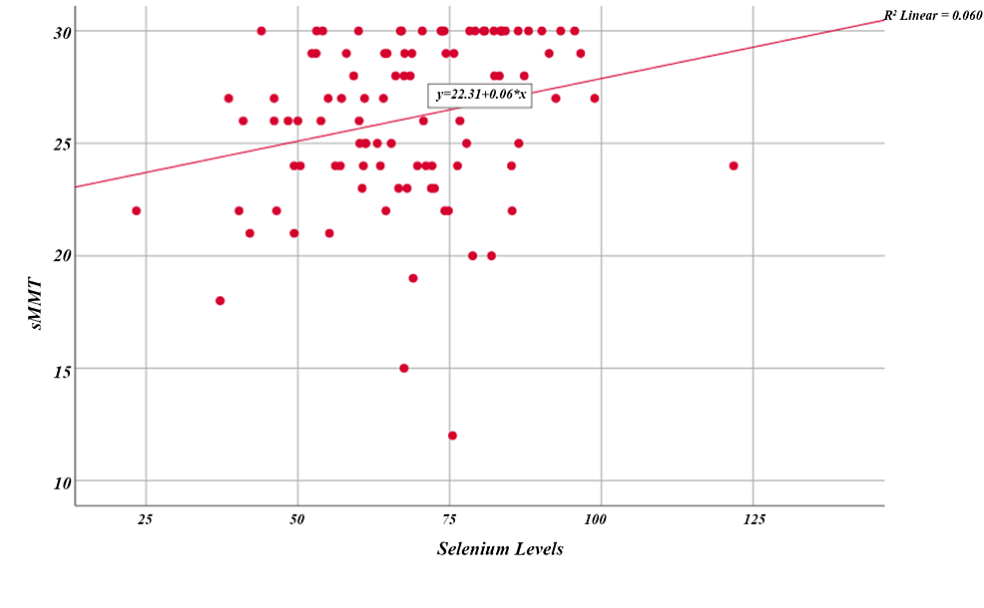
Relationship between sMMT score and serum selenium level sMMT: Standardized Mini Mental Test.

In correlation analysis, a statistically significant negative correlation was found between SSL and BDI scores (r=-0.301; p=0.002) (Table 4). A statistically significant negative correlation was found between BDI and sMMT scores (r=-0.359; p<0.001). A statistically significant positive correlation was found between SSL and sMMT scores (r=0.299; p=0.003). According to simple linear regression analysis, a 10-unit increase in SSL increased the sMMT score by 0.6 units (Figure 3).

**Table 4:** Results of correlation analysis between serum selenium level, BDI, and sMMT **p<0.01. SSL: serum selenium levels; BDI: Beck Depression Inventory; sMMT: Standardized Mini Mental Test.

Variables		BDI score	sMMT score
SSL	r	-0.301**	0.299**
	p	0.002	0.003
BDI	r		-0.359**
	p		<0.001

## Discussion

Cognitive impairment is commonly observed in CKD patients but is usually diagnosed after clinical progression since its importance is not known enough. Early diagnosis of cognitive dysfunction is crucial because it has negative effects on morbidity, mortality, and quality of life of the patients [16]. In one study, cognitive impairment, which was 2.9% in the initial evaluation of patients receiving HD treatment, was found to be 87.3% as a result of further and detailed evaluation, indicating that non-severe cognitive impairment is often overlooked [17]. Adding cognitive function assessment to routine examination in patients diagnosed with CKD may increase compliance with CKD treatment by providing early detection and management of cognitive impairment [18].

Cognitive impairment was detected in 20-87% of patients receiving HD treatment [19]. In one study, cognitive impairment was found in 28.7% of patients receiving PD treatment [20]. Another study observed that those who received PD treatment for ESRD had better cognitive functions than those who received HD treatment. The authors link this result to the fact that they may have selected patients with more cognitive function competence because the patients are required to participate actively in PD treatment. Concomitant diabetes, HT, dyslipidemia, anemia, and uremic toxins are thought to affect cognitive impairment [21]. In addition, it is stated that hypotensive attacks, cerebral microhemorrhages due to anticoagulation, and sudden electrolyte changes that can be seen during the HD session may cause more cognitive impairment in HD patients [22]. In our study, we excluded cases in which dialysis-related hypotension, changes in consciousness, and known cerebrovascular events exist. Even though similar to the literature, cognitive impairment was found in 30% of those who received dialysis treatment and in 16% of all CKD patients (n=75). Moreover, sMMT scores were found to be lower in HD, PD, and stage 3-4 CKD patients, respectively, and reached statistical significance. We think that the higher diagnosis of diabetes in CKD patients participating in the study in PD and stage 3-4 CKD patients may have led to this result.

Depression is very common in ESRD patients and is seen at a rate of 20-25% [23]. In addition, depression in ESRD patients has been associated with increased morbidity and mortality [24]. Similar to the literature, in this study, moderate or severe depression was detected in 44% of HD patients, 50% of PD patients, and 40% of stage 3-4 CKD patients. While BDI scores were statistically significantly higher in HD and PD groups than in controls, there was no statistically significant difference in BDI scores between stage 3-4 CKD and controls. When the participants were evaluated according to the BDI groups, it was seen that the group with the highest sMMT value was the minimal depression group, which shows that depression may affect cognitive functions.

Oxidative stress is known to play a role in neurodegenerative diseases such as Alzheimer's disease and cognitive impairment [25]. It is known that selenium in the structure of selenoproteins exhibits neuroprotective effects with its antioxidation property. Derbeneva et al. [4] found that selenium effectively improves cognitive functions, and dietary selenium intake is also low in people with low cognitive functions. In CKD patients, especially those receiving dialysis treatment for ESRD, oxidative stress was statistically significantly higher [26] and dialysis patients had lower SSD than healthy volunteers [5]. Some studies have emphasized that as the CKD stage progresses, the selenium level decreases [27], which may be related to the kidney proximal tubule epithelium’s effectiveness in the absorption of selenium [28]. In addition, it is stated that animal protein-restricted diets, one of the most important sources of selenium in these patients, may cause low selenium levels due to renal protein loss and related malnutrition [29].

In our study, SSLs of stage 3-4 CKD and control groups were statistically significantly higher than those of HD and PD groups. We also found a statistically significant positive correlation between SSL and sMMT scores. This finding suggests that selenium may be effective in preserving cognitive functions in CKD patients. In addition, a statistically significant negative correlation was found between SSL and BDI scores, which supports a relationship between low SSL and depression in the study population.

When the literature was examined, it was seen that the relationship between selenium and depression could not be clearly revealed before [30]. In some studies, it was found that low dietary selenium intake may be a high-risk factor for the development of major depression [7], but in some studies, no statistically significant correlation was found between low selenium and depression in HD patients [8]. Some studies have even suggested that high selenium exposure is associated with symptoms of severe depression [9]. It will be possible to obtain more precise results with future research on this subject.

Limitations

Our study has some limitations due to the design of the study; it has a relatively small sample size and a single ethnic origin. Also, it was a single-center study. However, the study group's specificity increases the study's power.

## Conclusions

Both cognitive impairment and depression are common in patients receiving dialysis treatment for ESRD, and depression may affect cognitive impairment. Selenium deficiency is correlated to the emergence of cognitive impairment and depression in dialysis patients. Investigation of the effects of selenium supplementation and increasing dietary selenium intake on cognitive functions and depression in patients with the impaired renal function will increase the strength of our findings.

## References

[1] Radić J, Ljutić D, Radić M, Kovaĉić V, Sain M, Curković KD (2010). The possible impact of dialysis modality on cognitive function in chronic dialysis patients. Neth J Med.

[2] Yıldız D, Seferoğlu M, Güneş A (2018). Assessment of cognitive dysfunction in hemodialysis patients by the mini mental test and the clock drawing test. Turkish J Nephrol.

[3] Torres DJ, Alfulaij N, Berry MJ (2021). Stress and the brain: An emerging role for selenium. Front Neurosci.

[4] Derbeneva SA, Bogdanov AR, Pogozheva AV, Gladyshev OA, Vasilevskaia LS, Zorin SN, Mazo VK (2012). Effect of diet enriched with selenium on the psycho-emotional and adaptive capacity of patients with cardiovascular diseases and obesity. Vopr Pitan.

[5] Turkmen K, Ecder T, Turk S (2012). Effects of serum selenium level on cell-mediated immunity and on antibody response to multivalent influenza vaccine in hemodialysis patients. Turkish J Nephrol.

[6] Fullerton CS, Ursano RJ, Wang L (2004). Acute stress disorder, posttraumatic stress disorder, and depression in disaster or rescue workers. Am J Psychiatry.

[7] Pasco JA, Jacka FN, Williams LJ (2012). Dietary selenium and major depression: A nested case-control study. Complement Ther Med.

[8] Ekramzadeh M, Mazloom Z, Sagheb M (2015). Association of depression with selenium deficiency and nutritional markers in the patients with end-stage renal disease on hemodialysis. J Ren Nutr.

[9] Colangelo LA, He K, Whooley MA, Daviglus ML, Morris S, Liu K (2014). Selenium exposure and depressive symptoms: The Coronary Artery Risk Development in Young Adults Trace Element Study. Neurotoxicology.

[10] Lam RW, Kennedy SH, Mclntyre RS, Khullar A (2014). Cognitive dysfunction in major depressive disorder: Effects on psychosocial functioning and implications for treatment. Can J Psychiatry.

[11] Stevens LA, Schmid CH, Greene T (2010). Comparative performance of the CKD Epidemiology Collaboration (CKD-EPI) and the Modification of Diet in Renal Disease (MDRD) Study equations for estimating GFR levels above 60 mL/min/1.73 m2. Am J Kidney Dis.

[12] Vacchina V, Dumont J (2018). Total selenium quantification in biological samples by inductively coupled plasma mass spectrometry (ICP-MS). Methods Mol Biol.

[13] Tombaugh TN, McIntyre NJ (1992). The mini-mental state examination: A comprehensive review. J Am Geriatr Soc.

[14] Turk S, Atalay H, Altintepe L (2006). Treatment with antidepressive drugs improved quality of life in chronic hemodialysis patients. Clin Nephrol.

[15] Beck AT, Ward CH, Mendelson M, Mock J, Erbaugh J (1961). An inventory for measuring depression. Arch Gen Psychiatry.

[16] Van Sandwijk MS, Ten Berge IJ, Majoie CB, Caan MW, De Sonneville LM, Van Gool WA, Bemelman FJ (2016). Cognitive changes in chronic kidney disease and after transplantation. Transplantation.

[17] Murray AM, Tupper DE, Knopman DS (2006). Cognitive impairment in hemodialysis patients is common. Neurology.

[18] Madero M, Gul A, Sarnak MJ (2008). Cognitive function in chronic kidney disease. Semin Dial.

[19] Saele K, Sønnesyn H, Svarstad E, Aarsland D (2009). [Cognitive failure in terminal kidney disease]. Tidsskr Nor Laegeforen.

[20] Shea YF, Lee MC, Mok MM, Chan FH, Chan TM (2019). Prevalence of cognitive impairment among peritoneal dialysis patients: A systematic review and meta-analysis. Clin Exp Nephrol.

[21] Drew DA, Weiner DE, Sarnak MJ (2019). Cognitive impairment in CKD: Pathophysiology, management, and prevention. Am J Kidney Dis.

[22] Drew DA, Tighiouart H, Scott TM, Lou KV, Shaffi K, Weiner DE, Sarnak MJ (2013). Cognitive performance before and during hemodialysis: A randomized cross-over trial. Nephron Clin Pract.

[23] Kimmel PL, Cukor D, Cohen SD, Peterson RA (2007). Depression in end-stage renal disease patients: A critical review. Adv Chronic Kidney Dis.

[24] Cohen SD, Norris L, Acquaviva K, Peterson RA, Kimmel PL (2007). Screening, diagnosis, and treatment of depression in patients with end-stage renal disease. Clin J Am Soc Nephrol.

[25] Tönnies E, Trushina E (2017). Oxidative stress, synaptic dysfunction, and Alzheimer's disease. J Alzheimers Dis.

[26] Ninić A, Sopić M, Munjas J (2018). Association between superoxide dismutase isoenzyme gene expression and total antioxidant status in patients with an end-stage renal disease. Balkan Med J.

[27] Zachara BA, Salak A, Koterska D, Manitius J, Wasowicz W (2004). Selenium and glutathione peroxidases in blood of patients with different stages of chronic renal failure. J Trace Elem Med Biol.

[28] Burk RF, Hill KE (2009). Selenoprotein P-expression, functions, and roles in mammals. Biochim Biophys Acta.

[29] de Luis D, Bustamante J (2008). Nutritional aspects in renal failure. Nefrologia.

[30] Wang J, Um P, Dickerman BA, Liu J (2018). Zinc, magnesium, selenium and depression: A review of the evidence, potential mechanisms and implications. Nutrients.

